# DeepFreeze 3D‐biofabrication for Bioengineering and Storage of Stem Cells in Thick and Large‐Scale Human Tissue Analogs

**DOI:** 10.1002/advs.202306683

**Published:** 2024-01-06

**Authors:** Alok Kumar, Robert A. Brown, Daniel Benyamien Roufaeil, Aditi Gupta, Erika L. Lipford, Divya Muthusamy, Amihai Zalzman, Ronna Hertzano, Tao Lowe, Joseph P. Stains, Michal Zalzman

**Affiliations:** ^1^ Department of Biochemistry and Molecular Biology University of Maryland School of Medicine Baltimore MD 21201 USA; ^2^ Cardiovascular Research Center Massachusetts General Hospital (MGH) Harvard Medical School Boston MA 02114 USA; ^3^ Department of Otorhinolaryngology‐Head and Neck Surgery University of Maryland School of Medicine Baltimore MD 21201 USA; ^4^ Department of Oral and Maxillofacial Surgery University of Maryland School of Dentistry Baltimore MD 21201 USA; ^5^ Fischell Department of Bioengineering University of Maryland A. James Clark School of Engineering College Park MD 20742 USA; ^6^ Department of Orthopedics University of Maryland School of Medicine Baltimore MD 21201 USA; ^7^ Department of Biochemistry and Molecular Biology Department of Otorhinolaryngology‐Head and Neck Surgery Marlene and Stewart Greenbaum Cancer Center The Center for Stem Cell Biology and Regenerative Medicine University of Maryland School of Medicine Baltimore MD 21201 USA; ^8^ Neurotology Branch NIDCD, NIH Bethesda Maryland United States

**Keywords:** 3D printing, 3D scaffolds, biofabrication, bioinks, regeneration, regenerative medicines, stem cells, tissue engineering

## Abstract

3D bioprinting holds great promise for meeting the increasing need for transplantable tissues and organs. However, slow printing, interlayer mixing, and the extended exposure of cells to non‐physiological conditions in thick structures still hinder clinical applications. Here the DeepFreeze‐3D (DF‐3D) procedure and bioink for creating multilayered human‐scale tissue mimetics is presented for the first time. The bioink is tailored to support stem cell viability, throughout the rapid freeform DF‐3D biofabrication process. While the printer nozzle is warmed to room temperature, each layer solidifies at contact with the stage (‐80 °C), or the subsequent layers, ensuring precise separation. After thawing, the encapsulated stem cells remain viable without interlayer mixing or delamination. The composed cell‐laden constructs can be cryogenically stored and thawed when needed. Moreover, it is shown that under inductive conditions the stem cells differentiate into bone‐like cells and grow for months after thawing, to form large tissue‐mimetics in the scale of centimeters. This is important, as this approach allows the generation and storage of tissue mimetics in the size and thickness of human tissues. Therefore, DF‐3D biofabrication opens new avenues for generating off‐the‐shelf human tissue analogs. It further holds the potential for regenerative treatments and for studying tissue pathologies caused by disease, tumor, or trauma.

## Graphical abstract

DeepFreeze‐3D biofabrication is a new procedure to generate large, well‐defined, human tissue analogs while avoiding interlayer mixing or delamination. This translational approach supports stem cells during rapid 3D printing under deep‐freezing conditions (‐80 °C) and opens groundbreaking avenues for creating functional off‐the‐shelf human‐scale tissues. Therefore, it has a great potential to impact regenerative treatments and the study of human pathologies.

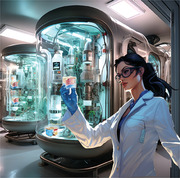

## 1 Introduction

Bioengineering of tissues is a promising approach for the development of therapeutic solutions for a wide range of debilitating diseases, such as organ failure and tissue damage. The ultimate goal is to create human tissue analogs with architectural complexity.^[^ ^1^, ^2^, ^3^ ^]^ However, significant challenges still remain in scaling up the generation of human tissue mimetics containing cells, into structures thicker than about one millimeter.^[^ ^4^, ^5^, ^6^ ^]^ Human cell incorporation into sizable and dense 3D structures exposes them to sub‐optimal conditions due to diffusion limits. Several factors affect stem cell survival and function, such as low oxygen supply, perturbations in pH equilibrium, and constrained access to vital nutrients within the core of the engineered tissue. Moreover, the extended duration of cross–linking, required for thick structures, exposes cells to chemicals or radiation for a prolonged time. Therefore, collaborative efforts from various scientific disciplines are required, to create functional tissues.

As tissues are composed of many cell types and layers, 3D structures can be biofabricated by casting into molds, or by 3D bioprinting using multiple printheads to create complex 3D shapes, using various bioinks (biomaterials).^[^ ^2^ ^]^ Likewise, different stem cells can be used to generate multiple tissue types.^[^ ^3^, ^4^, ^5^, ^6^ ^]^ The ideal bioink matrix should therefore not only dictate the tissue‐mimetic shape, but also provide stem cells with the optimized environment, and attachment platform, in the same way, they would attach to the matrix of normal tissues.

Different bioinks were reported, such as alginate‐gelatin‐carbon nanotube‐based bioink to make vascular grafts,^[^ ^7^ ^]^ alginate‐polyvinyl alcohol‐based bioinks for bone tissue ^[^ ^8^ ^]^ and alginate sulfate–nanocellulose‐based bioink for cartilage applications.^[^ ^9^ ^]^ Others rely on cross–linking by exposure to potentially cell‐transforming radiation such as UV light after printing. Some examples include gelatin‐methacryloyl‐alginate bioink for skeletal muscle,^[^ ^10^ ^]^ cell‐laden gelatin methacrylate,^[^ ^11^ ^]^ or Furfuryl gelatin‐based bioinks for cardiac applications.^[^ ^12^, ^13^ ^]^

Although advances have been made in creating thin 3D structures containing cells,^[^ ^14^ ^]^ current technologies still lack the ability to accurately create similar, large‐scale structures due to multiple challenges.^[^ ^15^, ^16^ ^]^ Further, mixing of the cell layers (interlayer mixing) during or after printing, compromises the microstructure and the cell function. This is even more challenging when attempting to create large‐bioengineered structures, as interlayer mixing, and maintaining microstructure fidelity becomes increasingly higher.^[^ ^15^, ^16^ ^]^ As we scale up the production of tissues and organs, the intricacies of preserving structural integrity and preventing undesired mixing between layers become vital concerns.

Here we report for the first time, DeepFreeze‐3D (DF‐3D) biofabrication, a novel procedure designed for the creation of large‐diameter, 3D tissue‐like structures on human scale. Our approach combines stem cells, customized biomaterials, and external cues to develop transformative solutions to creating mandibular bone tissues. DF‐3D‐printing and biofabrication are done under “deep freezing” conditions, also known as “low cryogenic” freezing at −80 °C. We show that DF‐3D biofabrication supports high stem cell viability during prolonged freezing, and function after thawing in cross–linking medium. The DF‐3D bioink‐encapsulated cells not only survive but also differentiate efficiently from bone cells throughout human‐size 3D scaffolds.

Our results demonstrate that DF‐3D biofabrication effectively maintains layer separation and supports stem cell viability when casted, 3D‐printed, or injected into 3D PLA scaffolds. Using this approach, we further show, that DF‐3D biofabrication can be used to generate large, thick, 3D‐tissue‐like structures in the scale of centimeters. Additionally, it further supports robust stem‐cell differentiation into bone cells, throughout the thickness of the tissue analogs, and after prolonged freezing and thawing.

## 2 Results and Discussion

The diffusion of oxygen and nutrients is restricted to about 200 µm, which poses challenges in creating 3D human‐size tissue mimetics. Consequently, cell‐laden 3D structures are restricted to less than a millimeter‐thick structures. To overcome these challenges, a low viscosity DeepFreeze‐3D (DF‐3D) bioink and procedure was developed, to support cell viability throughout the thickness of large structures, while being biofabricated at −80 °C. Alginate concentrations were optimized to serve as a base material, since it is biocompatible, bioinert, and can facilitate easy extrusion through very thin 3D printer nozzles. Collagen was added, to act as an extracellular‐matrix‐like substrate, and promote cell attachment, and adhesion. The physical properties of the bioink were enhanced by 1‐Ethyl‐3‐(3‐dimethylaminopropyl) carbodiimide, followed by incubation in N‐hydroxysuccinimide (EDC/NHS).^[^ ^17^ ^]^ Cells were added to the DF‐3D bioink (or control bioink), with a minimal cryoprotectant, for creating the desired 3D structures on a pre‐cooled −80 °C printer stage and stored at −80 °C until use. Finally, samples were thawed in a culture medium supplemented with CaCl_2_, at room temperature. This served as a second cross–linking step, to facilitate the linkage of alpha‐l‐guluronic acid (G‐blocks) of one alginate chain, with the G‐blocks of the adjacent chain through Ca^2+^. Control bioink samples without cryoprotectant, but otherwise identical composition, were prepared and cross–linked at room temperature.

For calibrating the cross–linking conditions, multipotent mesenchymal stem cells (MSCs) were tested, and found to tolerate incubation in up to 0.8 m CaCl_2_ in a culture medium for 10 min (Figure S1A, Supporting Information). As larger constructs require longer cross–linking, next, the cell viability was assessed following extended incubations in CaCl_2_ media. Our data show, that MSCs remain viable in well‐defined structures, after incubation for up to 5 h, at a final concentration of 0.05 m CaCl_2_ (Figure S1B, Supporting Information). Therefore, these conditions were used for further experiments, to allow the extension of incubation time, when needed, according to the sample dimensions.

### 2.1 DeepFreeze Casting of Low Viscosity Bioink, for Generation of Large 3D Structures

**Figure 1:**
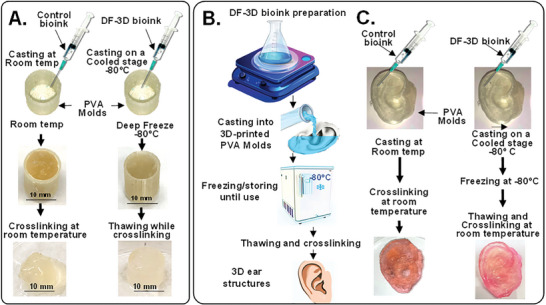
DeepFreeze 3D (DF‐3D) biofabrication of complex human‐size tissue‐like structures with high accuracy. A) Representative pictures of DF‐3D bioink casted into 3D printed cylindrical PVA molds at −80  C, compared to conventional alginate collagen bioink controls, casted at room temperature. The resultant control cylinders show surface irregularities and deformed structure (*n =* 6; left), compared to DF‐3D biofabricated at deep freeze temperatures (−80 °C) (*n =* 6; right). The DF‐3D biofabricated cylinders thawed in cross–linking solution at room temperature resulted in well‐defined structures without anomalies (bottom right). B) An illustration of casting bioinks into degradable PVA scaffolds of human‐size ear. C) Representative pictures of DF‐3D biofabricated (bottom right) or control Alginate‐collagen bioink (bottom left) human‐scale external ear structures. The DF‐3D biofabricated human ears show a well‐defined structure with superior shape fidelity (right), compared to the controls (left) showing structural deformation (*n =* 3 per group). All experiments were reproduced by three independent experiments.

To assess the structural stability of DeepFreeze‐3D (DF‐3D) bioink after thawing, water‐soluble Polyvinyl alcohol (PVA) cylinder molds were 3D printed (Ultimaker S5 3D printer), using fused deposition modeling (FDM). Next, DF‐3D bioink was casted into the PVA molds (*n =* 6) on a stage cooled to deep freeze temperatures (−80 °C). Then, the fully frozen structures were immersed and thawed in cross–linking medium, at room temperature for 10 min. As controls, another set of PVA cylinder molds were casted with bioink with similar composition, but without cryoprotective agent, and cross–linked after casting at room temperature (*n =* 6). As expected, surface irregularities and deformed structures were consistently observed in all the controls casted and cross–linked at room temperature (**Figure** **1**A, bottom left). Conversely, fabrication at deep freezing temperatures using DF‐3D bioink and thawing in cross–linking media, lead to a well‐defined cylindrical structure with a smooth surface (Figure 1A, bottom right).

Then, to assess the efficacy of generating large more complex structures, PVA molds of the full‐size human ear were 3D printed, and cast with control bioink (*n =* 3), or with DF‐3D bioink at −80 °C, and cross–linked (*n =* 3) (Figure 1B,C). Flat and deformed structures were formed from the control bioink with little resemblance to the original design (Figure 1C; bottom). Conversely, DF‐3D biofabrication led to solid ear‐like structures, with high accuracy, without surface anomalies or collapse (Figure 1C; bottom right).

### 2.2 Creating Complex, 3D Anatomical Architectures with High Fidelity

**Figure 2:**
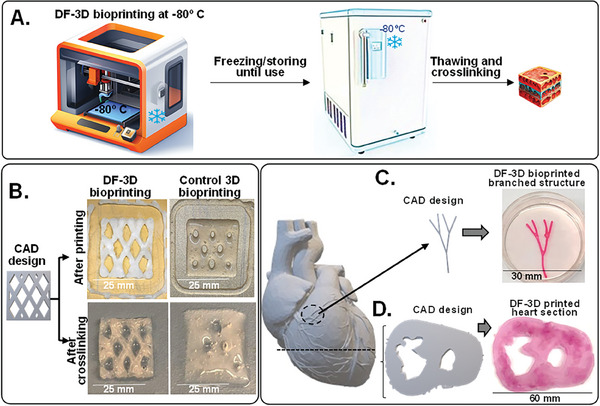
DeepFreeze 3D bioprinting of complex structures with high speed and accuracy. A) Schematic illustrations of DeepFreeze 3D (DF‐3D) bioprinting, deep freezing upon contact with a cooled stage at −80  C, and thawing in cross–linking solution. B) A CAD design of multilayered 25 × 25 mm, and 5 mm thick structures with diamond‐shaped porosity (left). The corresponding 3D structures were printed using either DF‐3D bioink on an −80  C cooled stage or with control bioink at room temperatures, each layer was 500 µm thick. The lower panel shows side‐by‐side representative photos immediately after printing (top) and after cross–linking (bottom). C) A CAD design and representative pictures of DF‐3D bioprinted high‐resolution (500 µm) vascular‐network‐like structures. D) A CAD model of a human heart and a slice through it (left), and the corresponding DF‐3D bioprinted heart section, printed at a speed of 10 mm s^‐1^.

To achieve accurate placement of multilayered architectures, DeepFreeze 3D‐bioprinter was developed, with a stage precooled to deep‐freezing conditions (−80 °C). As each layer of the bioink freezes upon contact with the printer stage, or with previous layers, DF‐3D biofabrication achieves precise layer separation and minimal bioink diffusion, resulting in well‐defined 3D structures (**Figure** **2**A). To first examine the process, a computer‐aided design (CAD) file was devised for rapid horizontal 3D bioprinting of a 2.5 cm^3^ square shape, with a diamond pattern (*n =* 3 per group) (Figure 2B; Left). As expected, 3D printing with the control bioink at room temperature leads to structural collapse during printing (Figure 2B; top right). Moreover, further structural deformation occurs after cross–linking, and the resultant shapes no longer resemble the original design (Figure 2B; bottom right). Conversely, DF‐3D biofabrication generated the correct size and thickness, with a well‐defined pattern and structure (Figure 2B; left). Next, to further validate the efficacy of creating complex structures, DF‐3D bioink was used to DF‐3D bioprinted branch‐like (mimicking‐vasculature) structures (25 mm long) (Figure 2C). As the 3D printer designed for these experiments has a 25‐gauge nozzle, the resultant diameter of each printed layer is 500 µm. These, for example, are smaller than the average thickness of veins, which range in size from <1 mm to 1.5 cm in diameter. In the future, we plan to design tissue‐like structures with narrower nozzles to test the resolution limit.

Finally, to demonstrate the ability to print large, complex anatomical architectures, a section of a human heart (depicting left and right ventricles) was DF‐3D bioprinted at 10 mm ^−1^s print rate using a CAD file, based on a digital design (Figure 2D). Following freezing at −80 °C and cross–linking for 10 min, a structural complexity was achieved, demonstrating detailed anatomical structures, with mechanical stability. This is a major advantage, over conventional biofabrication methods using viscous bioinks at room temperatures, as it allows for high‐speed bioprinting with high accuracy.

**Figure 3:**
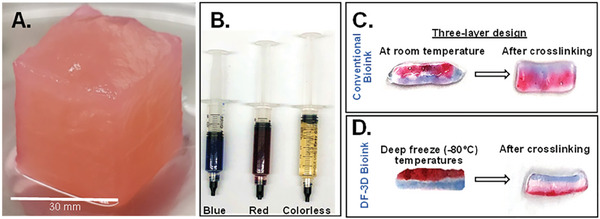
DF‐3D biofabrication of large and multilayered structures. A) DF‐3D bioink was casted into a 3 cm^3^ cubical PVA mold, kept in ‐80̊°C overnight, and thawed in cross–linking medium. PVA was removed and a stable 3 cm^3^ cubical shape was attained (*n =* 3). B) To evaluate interlayer mixing, DF‐3D Bioink (without cells) was dyed either in red (alizarin red), blue (hematoxylin), or left colorless. C) Using syringes, three‐layered 3D structures were hand printed at room temperature or in D) at ‐80̊ °C Samples were cross–linked and visually assessed for interlayer mixing (*n =* 6 per condition, in 3 independent experiments).

Furthermore, DF‐3D was also able to generate large‐scale, thick 30 mm^3^ structures, by casting DF‐3D bioink into PVA molds followed by storage at −80 °C. Thawing in cross–linking medium for 30 min led to a stable well‐defined structure (**Figure** **3**A). These data show the robustness of this approach, to yield stable structures from low‐viscosity bioink, in the thickness and accuracy needed for generating human‐scale structures.

### 2.3 DeepFreeze 3D‐Bioprinting of Multilayered Structures

A major challenge in designing 3D‐printed organs is the ability to create multilayered architectures. To evaluate the accuracy of generating multilayered structures, DF‐3D bioink was prepared (without cells) and dyed red, blue, or remained unstained (transparent) (Figure 3B). Then, using syringes, three‐layered structures were manually printed (*n =* 6), on a metal plate at deep freezing conditions. As controls, similar samples were printed at room temperature (Figure 3B). As expected, our results show that the 3 layers printed at room temperatures quickly diffused, lost structure form, and diffused even further during cross–linking, leading to deformed structures, with no clear layer separation (*n =* 3) (Figure 3C). Conversely, our DF‐3D results show that each layer freezes rapidly, and three layers remain well‐defined following cross–linking (*n =* 3) (Figure 3D). These results indicate that low‐viscosity DF‐3F bioink can be used for generating multilayered structures with minimal interlayer mixing and without delamination.

### 2.4 DF‐3D High‐Definition (HD) Multilayer‐Cell Printing Without Interlayer Mixing

**Figure 4:**
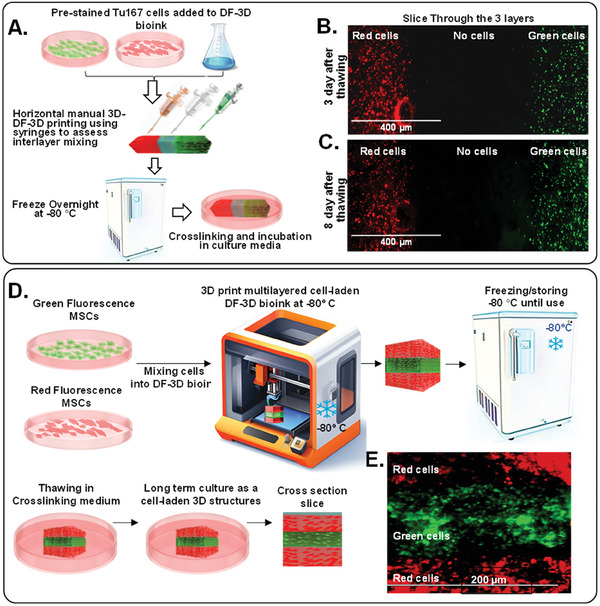
DF‐3D bioprinting for generating cell‐laden multilayered structures without microscopic interlayer mixing. A) A Schematic illustration of the stepwise design of 3 layers of DF‐3D bioink, preloaded with green or red cells, with a clear middle layer. B) Representative microscope picture of a section through the 3 layers of DF‐3D bioink indicating no mixing of cells at the interface layer: the first layer contains Tu167 cells prestained with CMTPX (Red; left), the middle layer is cell‐free, and the third layer contains Tu167 cells prestained with CMFDA (green; right). C) Representative images of the same cells after 8 days. D) An illustration of DF‐3D bioink mixed with either GFP Tu167 cells or with MSCs stained in red fluorescence and then DF‐3D bioprinted and stored at −80  C for 24 h, then, thawed in cross–linking medium and cross–sectioned. E) Representative microscope images of a cross–section of DF‐3D printed structures showing 3 layers of MSC (red) and GFP (green). Size Bars indicate 200 µm.

To evaluate interlayer mixing at the microscopic level, DF‐3D bioink or controls were prepared, and cross–linked with EDC‐NHS and pre‐stained Tu167 cells.^[^ ^18^ ^]^ were then added Then, syringes were used to manually print at room temperature, or in 3F‐3D conditions (**Figure** **4**A). Three layers were horizontally printed side‐by‐side: a layer of cells stained with red fluorescent dye (CMTPX), a second layer without cells, and a third layer of cells stained with green fluorescent dye (CMFDA). As expected, the structures printed with the control bioink at room temperature puddled and completely lost form. Excitingly, pictures taken by a fluorescence microscope (x4) clearly show 3 defined layers, 3 days after printing with DF‐3D bioink, with negligible mixing at the clear interface (Figure 4B). Furthermore, minimal to no cell mixing was observed after 8 days in culture (Figure 4C).

Then, to determine the outcome of 3D multilayer printing, MSCs were pre‐stained in red fluorescence (CMTPX; red), while Tu167 cells were stained in green fluorescence (CMFDA; green). Next, cells were incorporated into DF‐3D bioink, and loaded separately into the DF‐3D bioprinter. Three layers were then DF‐3D printed to further assess layer mixing (Figure 4D). The resultant structures were cryogenically stored for up to a month without significant change to cell viability (Figure 4D). Samples were thawed into cross–linking medium, washed, and grown until taken for sectioning (Figure 4E). Cross sections show 3 distinct layers at resolution of ≈100 µm, with high fidelity and negligible interlayer mixing (Figure 4F). These results demonstrate the efficacy of DF‐3D bioprinting to create complex, high‐definition, multilayered structures containing cells, with high precision at the microscopic level (*n*>3 per condition).

### 2.5 High Shape Fidelity, Printability, and Mechanical Stability of DF‐3D Bioprinted Structures

Bioink properties such as viscosity and storage modulus, directly affect the shape fidelity of 3D‐printed structures. Therefore, high‐viscosity bioinks are widely used to create structurally stable constructs for tissue engineering purposes.^[^ ^19^, ^20^, ^21^ ^]^ However, the shear stress and pressure required for 3D printing of thick bioinks, not only elevates cell stress but also increases cell death during and after printing. Moreover, this stress can potentially affect the ability of stem cells to differentiate correctly and form fully developed tissues post‐printing.

**Figure 5:**
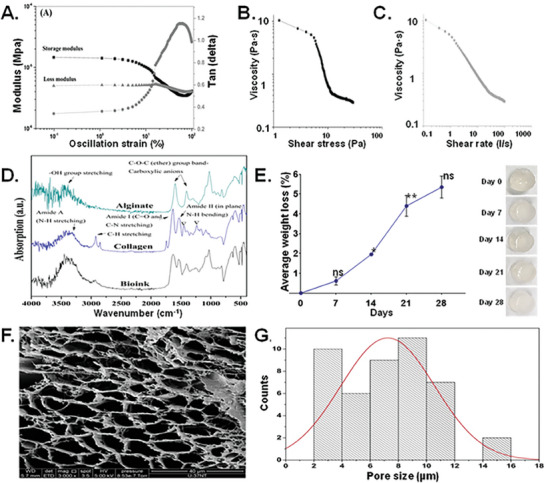
Mechanical properties, viscosity, yield strength, shape fidelity, and microstructure of DF‐3D bioink. A) The amplitude sweep confirms the viscoelastic nature of bioink with a higher storage modulus than the loss modulus. B,C) Shear stress versus viscosity curve shows the yielding behavior of viscosity with an increase in the shear stress, visible as a steep decrease in the viscosity at ≈ 5 Pa. D) FT‐IR of freeze‐dried DF‐3D bioink (cross–linked with 0.05 m CaCl_2_). Results confirmed the presence of alginate and collagen in the bioink in its pure form. The triple helical structure of the collagen was intact during the collagen gel preparation and bioink synthesis, confirmed by comparing the intensity of absorption peaks at 1235 cm‐1 and 1450 cm‐1. E) Dissolution curve long‐term dissolution study indicates a very slow biodegradability of the designed DF‐3D bioink with no abrupt increase in dissolution over 28 days. One‐way ANOVA with repeated measurements was used followed by multiple post hoc comparisons; * *p<*0.05, ** *p<*0.01, n.s ‐no significance. Photographs of the scaffolds (right), demonstrate structural integrity of the scaffolds. F) Representative DF‐3D ‐SEM pictures of scaffolds without cells showing porous structure. G) Size distribution analyses of DF‐3D bioink pores (µm).

The shape fidelity of bioinks is defined by their shape‐retention ability, which depends on the storage modulus and loss modulus. A higher storage modulus (*G*ˊ) represents more solid‐like properties, while a higher loss modulus (*G*˝) means more liquid‐like properties. Thus, a higher storage modulus improves mechanical strength by enhancing the ability of the hydrogel to store deformation energy and recover it in an elastic manner. The mechanical properties of our DF‐3D bioink were tested at room temperature, using a rheometer (**Figure** **5**A–C). The amplitude sweep provides information about the viscoelastic behavior of bioinks. The value to strain indicates at which point the bioink changes from a solid‐like phase to a more liquid‐like phase. The amplitude sweep at constant frequency shows a low value of storage and loss modulus (Figure 5A). This is advantageous, as the low modulus and viscosity of the DF‐3D bioink prior to cross–linking also increases the printability, reduces the extrusion pressure, and relieves the sheer stress imposed on cells during printing.

Then, to determine if DF‐3D bioink behaves like a gel (solid), or like a viscoelastic liquid, a frequency sweep test was carried out at room temperature (24 °C) at a 1%, fixed oscillation strain (Figure 5A). The storage modulus shows an increasing trend with increased frequency, while the Tan (delta), a ratio of loss modulus and storage modulus, increases at a lower frequency and then shows a decreasing trend of frequency, indicating a gel‐like behavior (Figure S2A,B, Supporting Information). These results show that our designed bioink holds form and structure. This is important, as while deep freezing allows for printing of additional layers to create larger structures, the gel behavior keeps the bioink shape long enough before cross–linking, Consequently, the cross–linking can be done during thawing.

To further study the printability of our bioink, a shear stress ramp was used to determine the yield stress of the bioink. The plot between shear stress and viscosity provides the point at which the DF‐3D bioink begins flowing during extrusion (Figure 5B). The results show a decrease in the viscosity with increased shear stress. At ≈1 Pa, the viscosity of the bioink was 10 Pa·s, which decreases linearly until ≈5 Pa. After that, a steep decrease in the viscosity was noted due to the yielding of the bioink. This is another advantage over viscous bioinks which require higher extrusion pressure, which causes cell stress during printing. Furthermore, compared to previously reported bioinks,^[^ ^22^ ^]^ DF‐3D bioink demonstrates a significantly lower value of yield stress (≈5 Pa), (70–200 Pa), and shear‐thinning behavior, characterized by a decrease in viscosity with increased shear rate (Figure 5C). These properties enable easier extrusion through narrower nozzles for the creation of detailed structures.

Structural resilience is required for safe handling and for stable tissue‐like constructs. Therefore, compression tests were performed on cylindrical structures of cross–linked DF‐3D bioink, at 37 °C in PBS using ISO 604:2002 (International Organization for Standardization) standard. Our results show a compressive strength of ≈2.520 ± 0.235 kPa which increases before fracture. Stress‐strain curve shows multiple yield points and an increase in the stress value after the fracture point (Figure S2B, Supporting Information). This increase can be attributed to densification as a result of the closure of pores at higher compression. The bulk elastic modulus of our bioink scaffolds was ≈0.069 kPa, and the yield strength (yield point) was also low ≈1.720 ± 0.143 kPa, which signifies the small elastic region, and thus the range in which the scaffolds can be compressed (Figure S2B, Supporting Information). Overall, the measured bulk elastic modulus of the bioink is in the range of soft tissues such as brain, fat, skin, and breast.^[^ ^23^, ^24^, ^25^ ^]^ These data demonstrate the high printability of DF‐3D bioink and further show it can be used to create mechanically stable structures with the intrinsic details needed for tissue engineering applications.

### 2.6 Composition Mechanical Testing Reveal Stable Structures with Interconnected Pores

The composition and porosity of scaffolds (including pore size and surface topography) can significantly affect the cell‐to‐material interactions, hence, their biocompatibility. Therefore, compositional and microstructural analyses of the scaffolds were performed. Using Fourier‐transform infrared spectroscopy (FTIR) analyses, DF‐3D bioink samples were compared with pure alginate and collagen powder (Figure 5D). As seen in the graph, absorption peaks at 3450, 1618, 1440, and 1050 cm^−1^ corresponds to ─OH group, ─COO─, ─COO─, and C─O, respectively, confirming the presence of alginate. Furthermore, the absorption peaks corresponding to Amide A (N─H stretching, 3225^,^ and 3280 cm^−1^), Amide I (C═O and C─N stretching, 1600 and 1700 cm^−1^), and Amides II (in‐plane N─H bending, 1510 cm^−1^ and 1580 cm^−1^) confirm the presence of collagen. The collagen is prepared by pepsin digestion at an acidic pH. Indeed, the results confirm a close to 1 ratio, in the intensity of absorption peaks at 1235 and 1450 cm^−1^, in both collagen and in our DF‐3D bioink, 1.015 and 1.146 respectively. These data demonstrate the integrity of the triple‐helical structure of the collagen in our DF‐3D bioink.

Then, we tested the degradation of our DF‐3D bioink, as ideally, it should be biodegradable and eventually be replaced by cells and tissues, To define the long‐term stability of DF‐3D structures at 37°C a swelling/dissolution assay was performed. As expected from an Alginate‐based bioink,^[^ ^26^ ^]^ the results show a negligible weight loss of the scaffolds over 21 days, and no significant swelling or change thereafter, representing low biodegradability (Figure 5E). These data were obtained from three biological replicates and reproduced in multiple independent experiments. The statistical significance between different time points was determined using one‐way ANOVA with multiple post hoc comparisons. Asterisks indicate a significant difference from previous time points. Importantly, no pH change was detected during the course of the study, which is beneficial, as a change in pH can harm cells incorporated in bioinks. Additionally, no significant dimensional changes in the scaffolds or swelling were observed, indicating structural stability (Figure 5E). Furthermore, data show no abrupt increase in dissolution over the course of 28 days, which indicates structural stability. These data indicate that 3D‐DF bioink prevents a burst release of cells or factors.^[^ ^27^ ^]^

Next, we performed a swelling study on freeze‐dried cylindrical‐shaped DF‐3D biofabricated scaffolds (10 mm diameter ׳ 9 mm length), in PBS at 37 °C. Data show a swelling (≈48 mg min^−1^) in the first 5 min, which then decreased to ≈3 mg min^−1^ in the next 5 min (Figure S3A, Supporting Information). After 10 min, no significant change in swelling was found. The average measured value of the equilibrium swelling ratio was 29.531±0.481 (mean ± SEM), and the percentage equilibrium water content was 96.723%±0.052.

Lastly, to determine the microstructure and porosity of DF‐3D hydrogel after cross–linking, a cryogenic‐scanning electron microscopy (cryo‐SEM) was performed. Data show porous structures (Figure 5F), with a pore size distribution range of 2–16 µm (Figure 5G), which is within the range radius of blood capillaries and may be useful in transporting oxygen and nutrients to encapsulated cells. To validate these data and exclude cryogenic freezing‐related artifacts, due to Cryo‐SEM, we prepared and cross–linked the DF‐3D bioink at room temperature. Then, the pore size of a thin section was assessed using a confocal microscope at 40× magnification. FIJI/ImageJ ^[^ ^28^ ^]^ analyses of the pores confirm the majority of interconnected pores size, ranging from 2 µm to 18 µm, with a mean of 10.67+/−38.8 (*n =* 265; *p* < 0.01). We noted that a few pores were significantly larger (18–100 µm), but these did not represent the overall consistency of the DF‐3D bioink (Figure S3B, Supporting Information). The absence of the larger pore size in the cryo‐SEM could be attributed to the focus on higher magnification (5000×). Overall, these data show that DF‐3D bioprinting yields stable 3D microstructure, with minimal dissolution, structural dimension changes, and biodegradability in prolonged culture.

### 2.7 DF‐3D Biofabrication Allows for Prolonged Cell Viability During Culture and Storage

**Figure 6:**
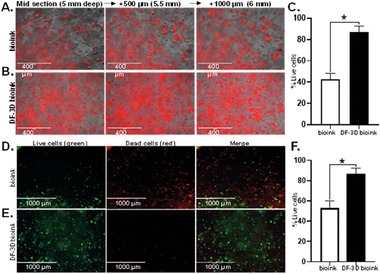
DF‐3D bioink protects cell viability in 3D structures. Representative microscope images of A) Control bioink cylinders containing MSCs stained with Cell tracker CMTPX dye (red) (*n =* 6) and B) equivalent DF‐3D biofabricated samples stained with CMTPX dye (red) demonstrate the presence and distribution of live cells. Pictures were taken from mid sections of a 1 × 1 cm (radius x height), at depth intervals of 500 microns from left to right. C. Quantification of live cells by red fluorescence using ImageJ shows a significantly higher cell viability in the DF‐3D bioink samples (*n =* 4, in 3 biological replicates) compared to the control bioink (*p <* 0.05). D) Representative pictures of Live/dead assay results in midsections of cell‐laden control bioink compared to E) cell‐laden DF‐3D bioink 24 h after thawing. Live cells are visualized in green and dead cells are in red. F) Quantification by ImageJ validating high cell death in the control bioink (*n =* 6). Conversely, high cell viability is demonstrated in the DF‐3D bioink samples (*n =* 6) after storage at −80 °C.

Most current bioprinting methods do not allow long‐term storage of large cell‐laden constructs. Cryopreservation is essential for clinical translation as it can allow the banking of off‐the‐shelf tissue replacements. To examine the effect of cryopreservation on the viability of multipotent mesenchymal stem cells (MSC), DF‐3D bioink containing unstained MSCs was casted into PVA molds and stored at −80°C for one week (*n =* 3 per group). To determine cell viability, frozen scaffolds were cross–linked, washed, and incubated in media at 37 °C, in a 5% CO_2_ humidified incubator. The PVA mold was then dissolved, and the media was changed to remove any residual PVA. For cell maintenance, the medium was replaced every three days. Control samples were prepared at room temperature, cross–linked, and henceforth treated the same as above. After 8 days, cells were visualized using Cell Tracker CMTPX dye which is converted into red fluorescence only in viable cells. The control bioink shows an average of 40% viable cells, while a homogenous distribution of viable MSCs was detected throughout the DF‐3D bioink matrix (**Figure** **6**A–C).

These data were further validated by Live/Dead viability assay. Here, green fluorescence is detected only in live cells, while dead cells are stained in red. The Quantification data show that while only 50% of viable cells survive throughout the layers of the control bioink, a high cell viability was seen in DF‐3D bioink (Figure 6D–F). Our data indicate that DF‐3D bioink protects the cell viability in 3D printed structures during and after cryogenic storage.

### 2.8 Efficient Osteoblasts Differentiation of Stem Cell After Thawing in Large DF‐3D structures

**Figure 7:**
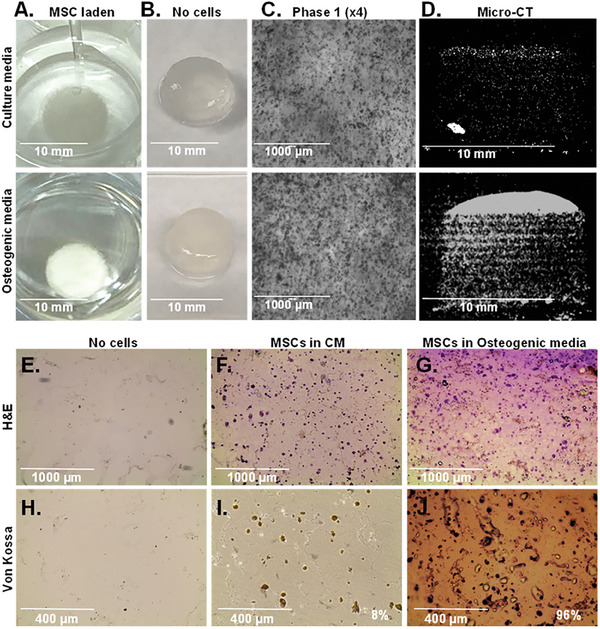
Efficient differentiation of MSCs to bone‐like tissue in DF‐3D cylinders. A. Representative photos of 10 × 10 mm MSC‐loaded DF‐3D bioink cylinders incubated in MSC‐culture medium (CM) (top), or osteogenic medium (bottom) for 35 days (*n =* 12 per condition). B. control samples without cells, at the indicated conditions. C. Representative phase 1 microscope images (4×) of equivalent scaffolds in CM, or osteogenic media. D. Representative vertical sections by Micro‐CT of the MSC‐laden DF‐3D bioink after 35 days in CM (top), or in osteogenic medium (bottom), showing mineralization. E. Representative images of H&E staining in horizontal mid‐section of DF‐3D biofabricated controls without cells or with, MSC in CM or G. osteogenic media for 5 weeks. Pictures show a tissue‐like structure (cytoplasm in pink and nuclei in purple). H) Von Kossa staining shows no mineralization in the control samples without cells or I)in MSC‐laden DF‐3D bioink after 35 days. J) High Von Kossa staining throughout the scaffolds after 5 weeks in osteogenic media (*n =* 6 per group).

To create functional tissues, cells must not only survive but, more importantly, differentiate efficiently. We have previously developed multipotent stem cells (MSCs) capable of differentiating into bone‐like cells.^[^ ^29^ ^]^ To evaluate the impact of DF‐3D bioprinting on the long‐term function of MSCs, we evaluated its effect on their osteogenic (bone) differentiation potential.^[^ ^29^, ^30^ ^]^ MSCs were incorporated into DF‐3D bioink at density of 2.9 × 10^6^ cells per 1 mL and 10 × 10 mm cylinders were generated and stored at −80 °C for 7 days (*n =* 24). The cell‐laden structures were then thawed in cross–linking medium for 10 min, washed, and cultured either in complete culture medium (CM), (*n =* 12) or in osteogenic (bone) differentiation medium (*n =* 12), for a total of 5 weeks. As additional controls, acellular scaffolds were used. Notably, the cell‐laden DF‐3D bioink incubated in an osteogenic medium showed visible white‐colored crystals (**Figure** **7**A, bottom), while the control samples remained unchanged (Figure 7A, top). The acellular controls in similar conditions remained transparent (Figure 7B). Phase microscopy assessment revealed a tissue‐like structure with high cell density in 3D space in both CM (Figure 7C, top) and osteogenic media (Figure 7C, bottom). Micro‐computed tomography (micro‐CT) analyses were performed to define the mineralization throughout the 3D scaffolds. As expected, our results show that the samples kept in MSC culture media had minimal mineralization (Figure 7D, top). However, the samples were grown in osteogenic (bone) differentiation media and presented with significant mineralization (Figure 7D, bottom). No mineralization was observed in the control samples without cells.

To assess the extent of bone cell differentiation within the 3D scaffolds, cryostat 10 µm cross sections were performed (*n =* 3 per group). Additional controls also included DF‐3D bioink samples without cells (*n =* 3). Then, H&E staining of cryosections in acellular DF‐3D bioink controls shows no cells present (Figure 7E), while a homogeneous distribution of cells (cytoplasm is stained in pink and nucleus in purple) was seen throughout the scaffolds grown in culture media (Figure 7F). A phenotype change was noted in osteogenic media with a tissue‐like structure, compared to cells grown in the MSC culture media (Figure 7G).

Next, to assess osteoblasts mineralization, Von‐Kossa staining was performed. As expected, no staining was seen in the empty scaffolds (Figure 7H). Light brown Von‐Kossa staining suggests some spontaneous differentiation in 6% of the MSCs in DF‐3D bioink, incubated in a complete medium (CM) (Figure 7I). Conversely, MSC‐containing DF‐3D bioink incubated in osteogenic medium for 5 weeks, shows a dark brown/black color staining in 96% of the cells (Figure 7J), suggesting a high density of calcium phosphate mineralization.

**Figure 8:**
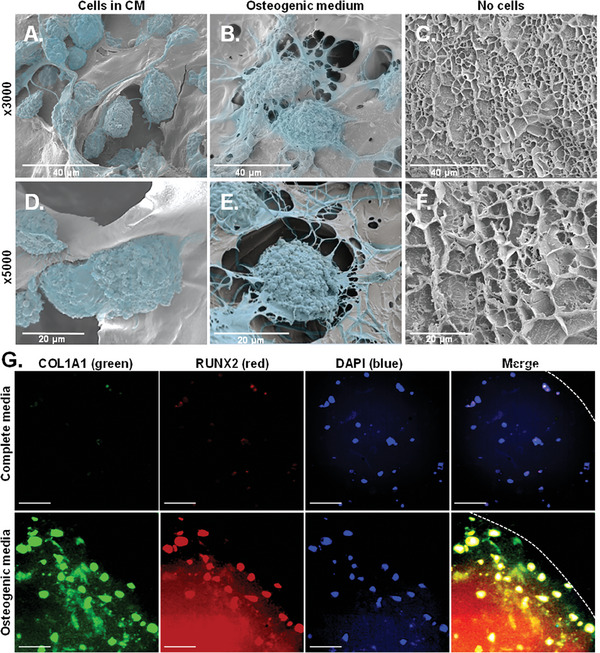
Bone‐like cell phenotype in DF‐3D bioink. Cryo‐scanning electron microscope (Cryo‐SEM) images (at 3000×, top; 5000×, bottom) of MSC‐laden DF‐3D biofabricated samples grown for 70 days in A) CM, or in B) osteogenic medium. C) DF‐3D bioink without cells grown in osteogenic media. D–F) High magnification cryo‐SEM images (5000×) show osteoblast‐like cells after differentiation when compared with control MSCs grown in CM. Cells are visualized in the pseudo color blue. G) Representative images of immunofluorescence staining of cross–section in the middle of the scaffolds (1 cm radius × 1 cm thick) grown in complete medium (top), or in osteogenic differentiation medium (bottom), with the bone cell markers RUNX2 and COL1A1. Nuclei are visualized by DAPI. Size bar indicates 50 µm.

To assess the microstructure of DF‐3D bioink containing differentiated cells, and control cells grown in CM, a mid‐horizontal section was performed on the scaffolds, and slices were taken to cryogenic scanning electron microscopy (Cryo‐SEM). Cryo‐SEM imaging was performed to determine the phenotype of the cells and visualize their interaction with the DF‐3D bioink. Cryo‐SEM images from the cell‐laden scaffolds grown in CM (**Figure** **8**A), or osteogenic medium (Figure 8B), reveal the presence of cells attached to the bioink matrix throughout both samples. DF‐3D biofabricated samples without cells grown in the osteogenic media served as additional controls (Figure 8C,F). Notably, both magnifications (3000×, 5000×) revealed, that while cells in CM maintained a fibroblast morphology (Figure 8D), the phenotype of cells cultured in an osteogenic medium dramatically changed to osteoblast‐like cells with spreading filipodia and mineralization (Figure 8E), further suggesting differentiation into osteoblasts. Finally, co‐immunostaining of MSC incorporated DF‐3D bioink and grown in an osteogenic medium, revealed the osteoblast‐specific markers RUNX2 (green) and COL1A1 (red) (Figure 8G), while the CM control samples remained negative. Taken together, these data indicate that DF‐3D biofabrication supports high stem cell viability and function, and efficient differentiation into bone cells after thawing and following a prolonged culture.

### 2.9 DF‐3D Bioink Supports Stem Cell Function in Composite Human‐Scale Mandibular Scaffolds

**Figure 9:**
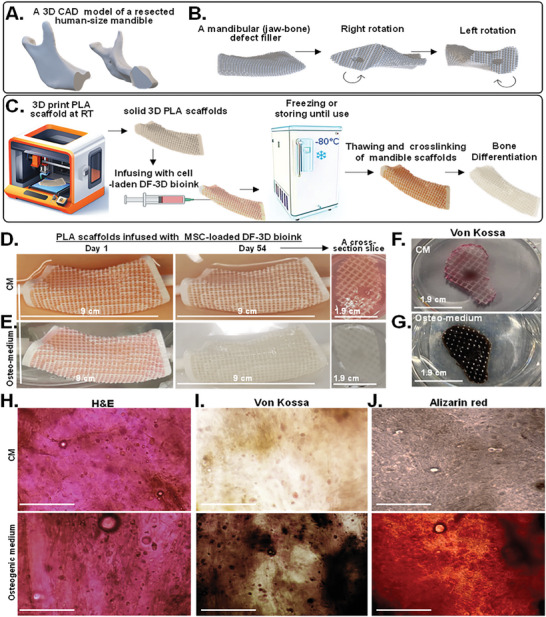
Composite human‐scale mandibular (jawbone) defect fillers incubated for 54 days show DF‐3D bioink supports an even distribution, high stem cell viability, and efficient differentiation into bone‐like cells throughout the scaffolds. A) A 3D CAD model of a full‐size human mandible (lower jawbone, without the teeth) after a 9 cm bone resection. B) The same structure rotated left or right to show the edges of each side. C) Illustration of generating composite full‐size human mandibular defect fillers. PLA scaffolds are 3D printed and infused with MSC‐laden DF‐3D‐bioink, frozen and −80 °C, thawed in cross–linking medium, and grown in culture. D) Representative pictures and cross sections of porous PLA scaffolds infused with multipotent stem cells (MSCs) in DF‐3D bioink and grown for 54 days in culture medium (CM) or E) in osteogenic medium. F) Representative photos of similar cross sections dyed with Von Kossa staining in CM or G) in osteogenic medium for 54 days. H) Representing microscope images from cross sections stained with H&E (*n =* 4 per group). I) Von Kossa (dark brown; (*n =* 3 per group), or J) Alizarin red (dark orange) (*n =* 3 per group), show bone tissue‐like morphology after 54 days in differentiation medium, but not in CM. Size‐bar = 100 µm.

Current bioinks still require means to promote cell survival and differentiation in scaffolds thicker than a millimeter.^[^ ^31^, ^32^, ^33^ ^]^ Limited supply of nutrients and oxygen in large 3D structures further leads to cell migration toward the periphery. Therefore, the viability and bone differentiation of MSCs embedded in human‐size scaffolds were assessed. Considering that DF‐3D bioink has a storage modulus within the range of soft tissues, and bone is mechanically stiffer than any hydrogel, 3D‐polylactic acid (PLA), was selected to generate composite structures. PLA is FDA‐approved for many applications and has the compressive strength necessary for the reconstruction of bone defects. It is likewise, slowly bioabsorbable, therefore, can eventually be replaced by native bone tissue. Using a CT scan, a computer‐aided design (CAD), of a human‐size mandible (lower jawbone), was generated and simulated a 90 mm non‐union fracture, similar to a defect created after a bone resection surgery (**Figure** **9**A). Then, the corresponding 90 mm long and 19 mm thick 3D‐mandibular defect filler was printed with PLA, using an FDM‐3D printer. The mandibular bone was designed with a middle channel and interconnected pores to allow even distribution (Figure 9B). The MSC‐laden DF‐3D bioink was infused through the middle channel, to be dispersed throughout the scaffolds. Next, scaffolds were kept at −80 °C for 24 h (Figure 9C). Additional controls were prepared, however, these could not be further used, as the bioink leaked through the pores at room temperature. Finally, to assess the differentiation efficiency, scaffolds were thawed, cross–linked, and cultured in CM (Figure 9D), or osteogenic medium (Figure 9E). Excitingly, after 54 days, no change was seen in CM, while the devices in the osteogenic medium became opaque. This was further seen in cross–section throughout the scaffolds (*n =* 12 per condition) (Figure 9E, right).

As cells were embedded in 19 mm‐thick scaffolds in the size of a human jawbone (Figure S4A–F, Supporting Information), to further visualize MSC distribution, cross slices were taken (10 mm and 40–50 mm of the mandible edge) (Figure 9F–J; Figure S4 G–J, Supporting Information). H&E staining of the cross sections (*n =* 4 per group) showed a homogenous distribution of cells throughout the scaffolds and a tissue‐like phenotype in both CM and osteogenic medium (Figure 9H). Strikingly, while Von‐Kossa staining reveals a sporadic light background in CM (Figure 9F; Figure S4G,H, Supporting Information), a high‐density stain was seen throughout the scaffolds in osteogenic conditions (*n =* 3 per group) (Figure 9G; Figure S4I,J, Supporting Information). These findings were supported by Alizarin red staining (Figure 9J), showing efficient bone differentiation in the depth of the human‐scale scaffolds.

**Figure 10:**
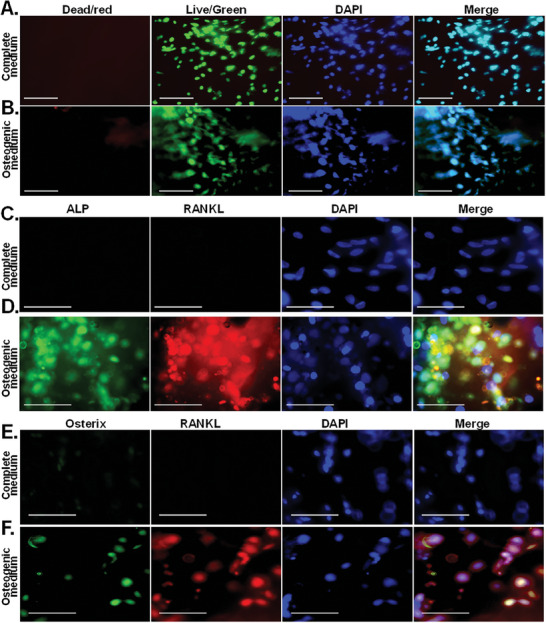
Long‐term MSC survival and bone differentiation‐markers in DF‐3D/PLA composite human‐size mandibular defect fillers. A) Representative images of live/Dead assays showing that DF‐3D bioink supports high cell viability (green) in the 54‐day experiment in CM and osteogenic medium in cross sections of human‐size mandibular defect fillers. Nuclei are visualized by Hoechst 33342 (blue). Size bars = 50 µm. C,D) Co‐immunofluorescence staining in cross sections taken from the middle of the 90 mm × 19 mm mandibular defect filler composite scaffolds: of the bone markers ALP and RANKL, or E‐F Osterix show positive staining only in MSCs grown in osteogenic medium, but not in CM. Nuclei are visualized by DAPI. Size bars = 50 µm.

Then, to test cell viability, Live/Dead staining was performed (*n =* 3 cross–sections per group). Our data show viable cells in both conditions, with similar distribution, demonstrating efficient survival of MSCs throughout the scaffolds with no noted limitation or preference for the periphery of the scaffolds (**Figure** **10**A).

Finally, to further evaluate cell differentiation in cross sections of the mandibular defect fillers, co‐immunofluorescence staining was used with the osteoblast markers ALP and RANKL, which were further complemented with co‐staining of Osterix and RANKL. Data show bright positive co‐staining of ALP, RANKL (Figure 10C,D), and Osterix with RANKL (Figure 10E,F) in the MSC‐laden DF‐3D biofabricated scaffolds, incubated in osteoblast medium while the control samples in CM, remained negative. These exciting results and comprehensive data, demonstrate that DF‐3D bioink allows efficient and effective cell survival and differentiation in large, human‐scale scaffolds.

## 3 Conclusion

In this work, we report for the first time DF‐3D biofabrication, a novel approach for creating viable, multilayered tissue mimetics in the scale and thickness of human tissues. DF‐3D bioengineering achieved high‐definition 3D printing to generate multilayer layers at a resolution of 150 µm. It further provides a major innovation as it allows the generation of large cell‐laden human tissue mimetics. Our data further show that this approach maintains the viability and function of stem cells during 3D printing, prolonged freezing, thawing, and culture for months. Not only that, but our stem cells thrive and differentiate to form bone tissue in human‐scale, large, 1.9 cm × 9 cm scaffolds. This is a major breakthrough compared to previously reported methods.

Cell‐loaded 3D constructs can be achieved by using high‐viscosity bioinks.^[^ ^34^, ^35^, ^36^, ^37^, ^38^ ^]^ However, the high viscosity creates challenges, such as clogging narrow printer heads, reducing printing speed, and requiring increased pressure for extruding the bioink during the printing process.^[^ ^39^, ^40^ ^]^ This is problematic because, as a result, any embedded cells are exposed to high shear stress and must spend a long time in suboptimal conditions, leading to reduced cell viability and function.^[^ ^34^ ^]^ When using stem cells, this issue becomes critical, as the inner, deep segments may remain devoid of cells. Furthermore, the suboptimal environment due to reduced oxygen and nutrients, as well as clearance of toxic substances, may hinder the viability and differentiation of stem cells into the desired tissue in large structures. Consequently, previously reported cell‐loaded 3D models, are still limited to millimeters.^[^ ^41^ ^]^

Conversely, bioinks with low viscosity can be used, to minimize the shear stress on cells and allow for faster printing. Yet, this comes at the expense of the final structural stability and resolution.^[^ ^42^ ^]^ A layer‐by‐layer cross–linking, likewise, slows down the printing process, leading to reduced cell viability and long‐term function. To resolve this, different methods, such as “Cryogenic 3D bioprinting” were developed to 3D print soft hydrogels at temperatures ranging from 0 °C to −20 °C.^[^ ^43^, ^44^ ^]^ A Cryo‐3D printing without cells, in an isopropanol bath, was reported, yet, these conditions are toxic and not suited for printing cells.^[^ ^45^ ^]^ Liquid nitrogen can be used to freeze a soft bioink while being extruded from a 3D printer. However, this is not suitable for stem‐cell encapsulation either, due to the use of rapid freezing.^[^ ^46^ ^]^ Lastly. a recent method was reported for vertical extrusion on a plate cooled to −20 °C. This approach generates small vertical bio‐structures made of fibers, such as muscle tissue. However, this method is limited to generating thin, short fibers with a diameter range of 0.5 to ≈ 1.25 mm.^[^ ^43^ ^]^ Therefore, new approaches are needed to create multilayered, large human tissue analogs.

Our DF‐3D approach holds multiple advantages: a). DF‐3D bioink has low viscosity and storage modulus utilizing a low concentration of cryoprotectant. This minimizes the extrusion pressure needed, allows for fast printing, and thereby reduces cell damage. b) The 3D printer stage is precooled to deep freezing conditions (−80 °C), while the 3D printer nozzle is heated to prevent clogging during printing. Therefore, as the layers freeze during printing, different cell types can be printed side‐by‐side and layer by layer, prior to cross–linking, which is essential for developing tissue mimetics. c) We optimized the procedure to utilize a low concentration of a non‐toxic cross–linker in culture medium, which is added in a single step upon thawing. d) DF‐3D allows long‐term cryogenic storage and transportation of cell‐laden structures.

Notably, other approaches limit the thickness of cell‐laden scaffolds to about a millimeter, as cells die at the core of scaffolds, or migrate toward the periphery, resulting in a very thin layer of viable cells at the outer layer of large scaffolds.^[^ ^1^, ^33^, ^43^, ^47^, ^48^, ^49^, ^50^ ^]^ Remarkably, our data indicate that DF‐3D bioink promotes efficient flow of nutrients, and gas exchange (CO_2_, oxygen), growth, and differentiation factors into the core of the scaffolds. We show efficient Stem cell differentiation throughout the thickness (1.9 cm × 9 cm) of human‐mandibular defect fillers (Figures 9 and 10; Figure S4, Supporting Information). Therefore, our approach represents a major leap in biofabrication of tissues, as it allows the survival and function of stem cells in large structures (90 mm by 19 mm) and provides an attachment surface for cells.

As cells can be cryopreserved in DF‐3D bioink, they can be used to generate off‐the‐shelf human tissue analogs for future regenerative therapies. The composition of DF‐3D can be bioink can be tailored to support different cell types and specific tissue applications. Therefore, the future development of this technology could have a far‐reaching impact on the creation of a wide range of tissues, such as heart, lung, kidney, liver, and cartilage. This may also provide a significant step toward the generation of tissues for drug testing and research purposes, potentially reducing the use of animal models. Furthermore, it may facilitate the creation, cryogenic storage, and transportation of off‐the‐shelf clinical‐size grafts for reconstructive surgeries.

## Conflict of Interest

The authors declare no conflict of interest.

## Supporting information

Supporting Information

## Data Availability

The data that support the findings of this study are available from the corresponding author upon reasonable request.
