# Supplementary material for: DeepFreeze 3D‐biofabrication for Bioengineering and Storage of Stem Cells in Thick and Large‐Scale Human Tissue Analogs
Source: Adv Sci (Weinh). 2024 Jan 6;11(11):2306683. doi: 10.1002/advs.202306683 (PMC10953591; doi:10.1002/advs.202306683)
Supplement: Supplementary file 1 — Supporting Information [file ADVS-11-2306683-s001.pdf]

ADVANCED SCIENCE
Open Access

Supporting Information

for *Adv. Sci.*, DOI 10.1002/advs.202306683

DeepFreeze 3D-biofabrication for Bioengineering and Storage of Stem Cells in Thick and Large-Scale Human Tissue Analogs

*Alok Kumar, Robert A. Brown, Daniel Benyamien Roufaeil, Aditi Gupta, Erika L. Lipford, Divya Muthusamy, Amihai Zalzman, Ronna Hertzano, Tao Lowe, Joseph P. Stains and Michal Zalzman**

Supplement Figure 1

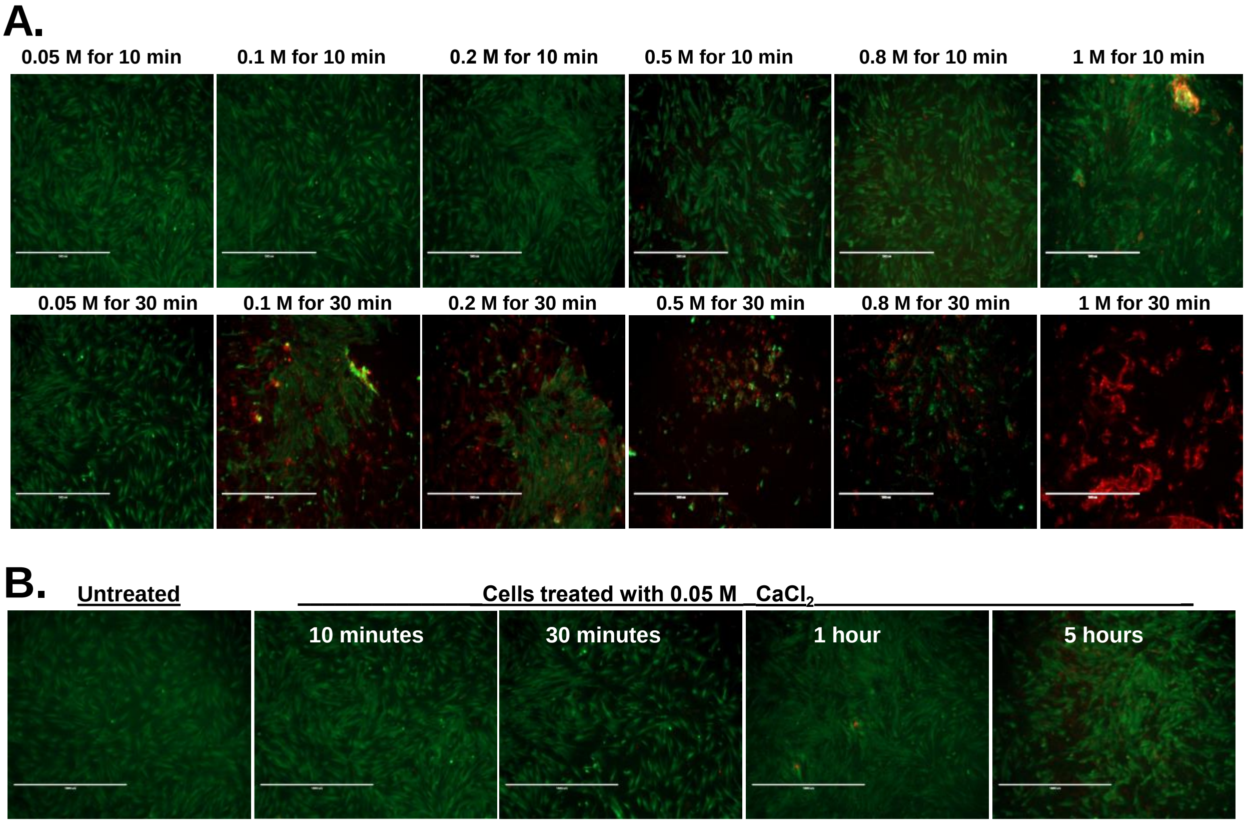

Supplemental Figure 1: Optimization of $CaCl_2$ concentration and incubation time. **A**. Representative images of MSCs incubated with increasing concentrations of $CaCl_2$ for 10 or 30 minutes. Cells were then stained with Fluorescence LIVE/DEAD™ cell staining (Invitrogen), marking live (green) and dead cells (red). The results indicate that our MSCs can tolerate culture medium containing a final concentration of 0.05M of $CaCl_2$ for 30 minutes without significant cell death. (n = 3 per condition). **B**. Subsequent experiment with MSCs show minimal cell death for up to 5 hours of incubation with 0.05M $CaCl_2$, which allows longer incubation of large structure when needed. n = 3 per condition in multiple independent experiments.

Supplement Figure 2

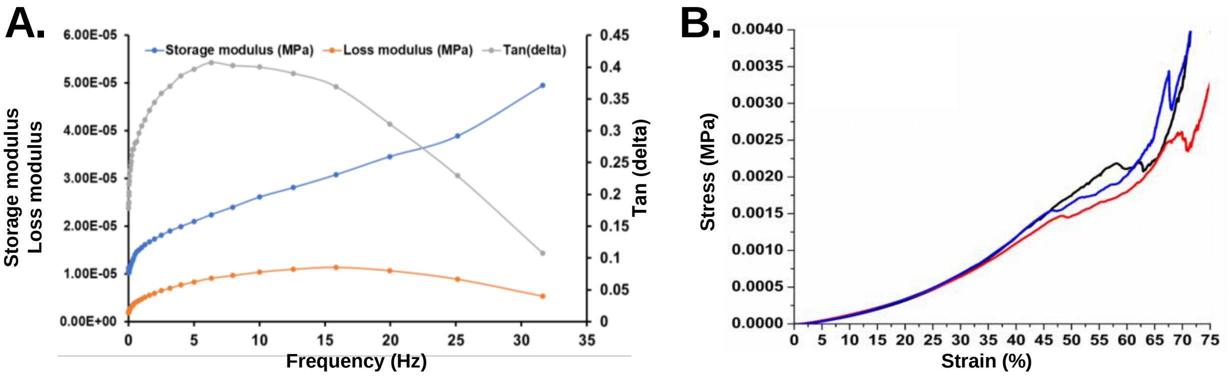

Supplementary Figure 2: Mechanical testing of DF-3D bioink. A. Frequency sweep of DF-3D bioink at room temp (22º C). DF-3D bioink show a dominating storage modulus over loss modulus across frequencies ranging from ~0 to ~31 Hz. A frequency-dependent increase in storage and loss modulus shows its viscoelastic property. These data indicate our DF-3D bioink have gel-like properties. **B**. Stress-strain curve showing an increase in the stress value during the compression test. An elastic region and yielding before fracture is clearly visible in the curve. An increase in the stress after fracture is due to the closure of pores during mechanical deformation, and therefore increase density of samples. This experiment was repeated 3 times, in multiple independent experiments.

Supplement Figure 3

A.

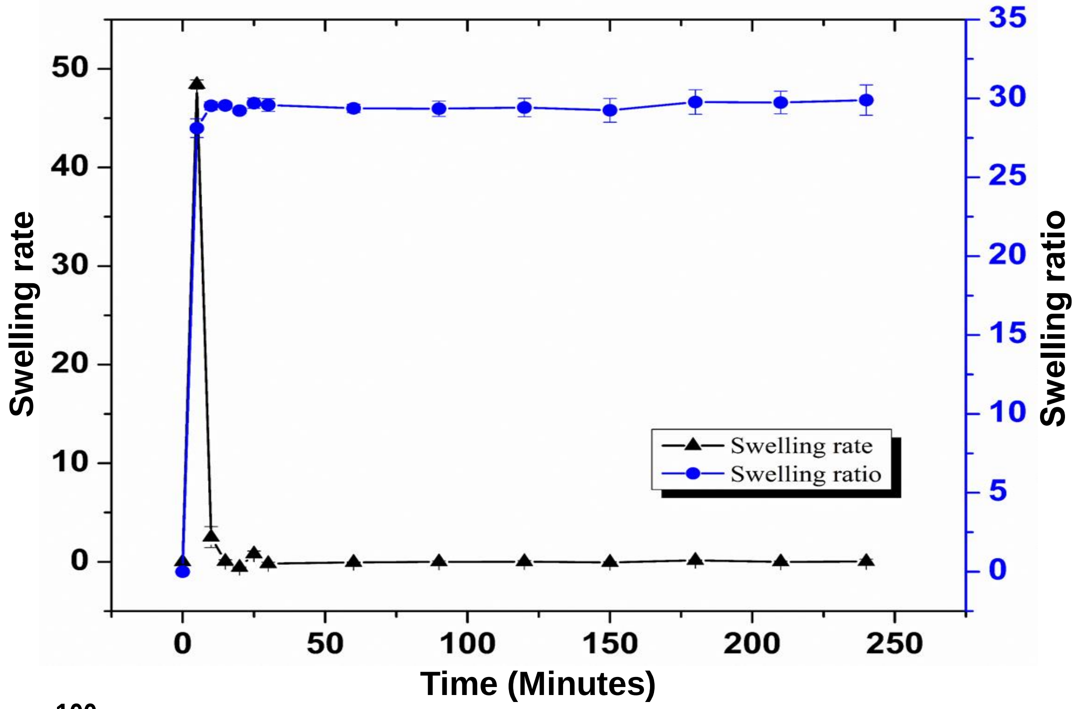

B.

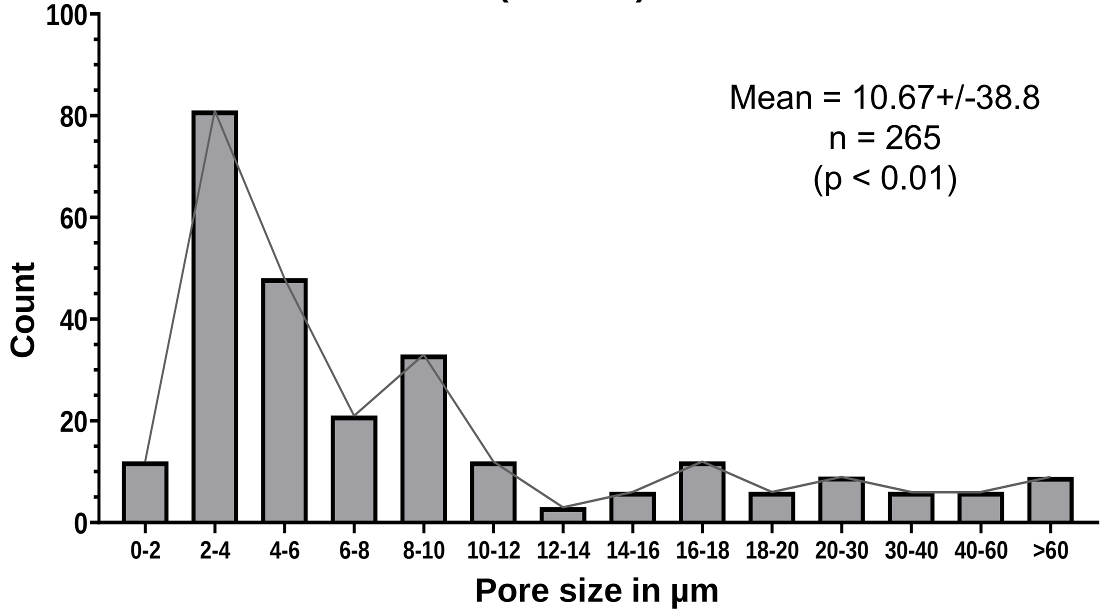

Supplement Figure 3: Mechanical testing of DF-3D bioink. A. DF-3D bioink samples (10 mm × 5 mm) were crosslinked and freeze-dried. Curves show the change in swelling ratio (marked with a solid circle) and swelling rate (marked with a solid triangle) after immersion in PBS. Weight was measured in multiple time intervals until a swelling equilibrium was reached. Swelling was noted in the first 10 minutes with increased swelling rate. After 10 minutes, equilibrium was reached with no further increase in swelling. **B**. DF-3D bioink pores size was assessed using a confocal microscope at x40 magnification. FIJI/ImageJ [47] analyses show that the majority of the

interconnected pores size range from 2 µm to 18 µm, with a mean of 10.67+/-38.8 µm (n = 265; p < 0.01). This experiment was repeated multiple times in independent experiments.

Supplement Figure 4

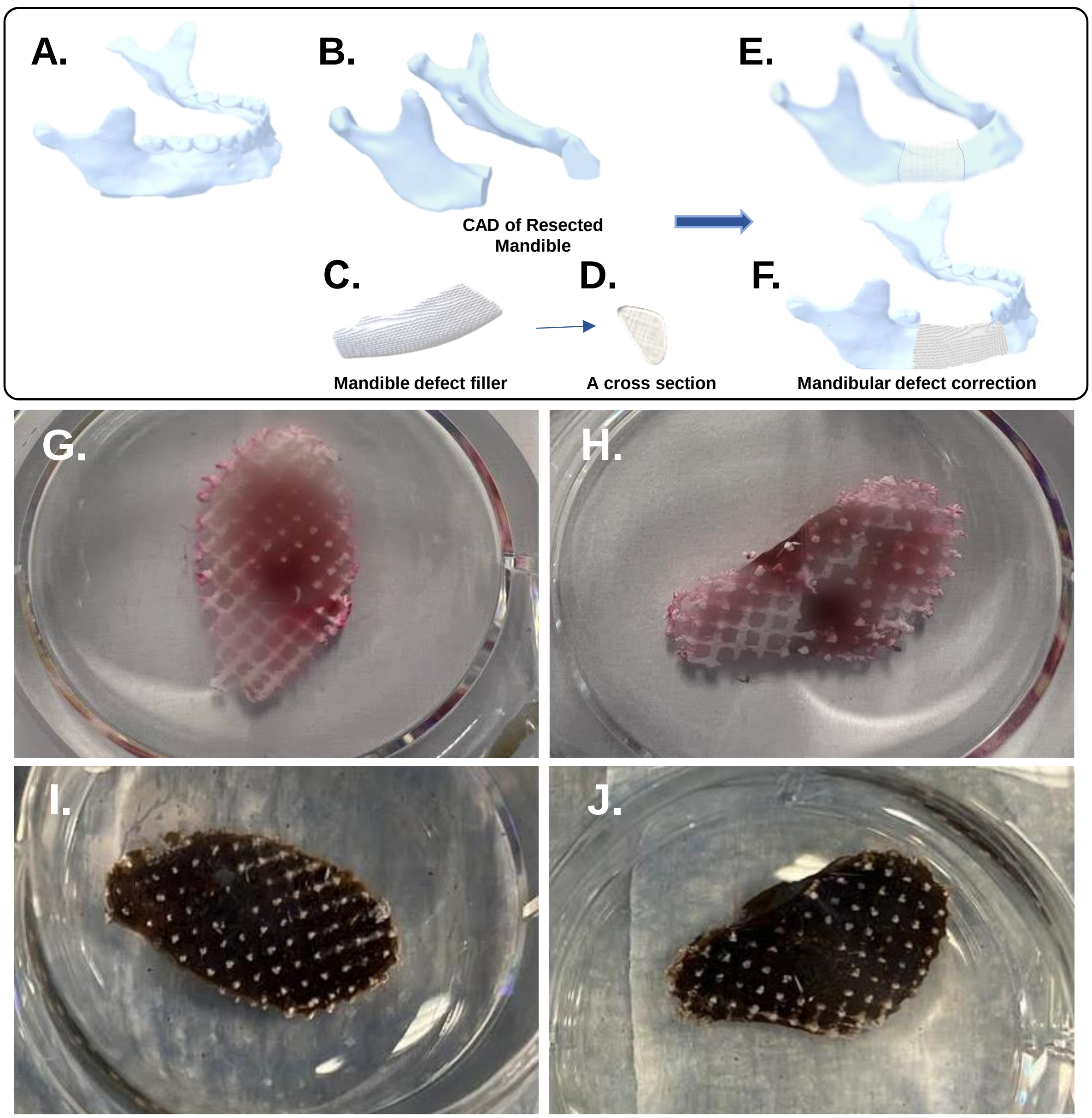

Supplementary Figure 4: DF-3D bioink facilitate efficient stem cell distribution and differentiation to bone in large human-scale devices. **A**, CAD was used to create a porous scaffold based on a CT scan of a human mandible (lower jawbone). **B**. Simulation of a 9 cm mandibular bone resection (with teeth omitted). **C**. The corresponding bone filler and **D**. A cross section of the bone filler. **E-F**. Simulation of insertion of the bone filler back into the resected bone with and without teeth. **G-H**. Additional photos of Von Kossa staining of cross sections (n = 3) preformed in the corresponding 3D printed PLA scaffolds, (also shown in **Figure 8**). These

scaffolds were infused with MSC-laden DF-3D bioink and grown in complete MSC culture medium, or **I-J**. in osteogenic medium for 54 days (n = 3 per group).

Supporting Information

Materials and Methods

Collagen and Alginate solutions

Alginate is an anionic polysaccharide, and a linear copolymer of β-(1-4)-linked D-mannuronic acid and β-(1-4)-linked L-guluronic acids. Stock solution of 2 wt.% alginate was prepared by dissolving 0.2 gr of alginate in 10 ml of sterile deionized (DI) water at room temperature and stirred overnight at 300 rpm. To make 3% collagen, first 1% collagen was prepared and then the volume was reduced to one third by centrifugation for 10 min at 4500 rpm. To make 1% collagen peptides, 15 mg pepsin was dissolved in 15 ml of 10 mM HCl at room temperature, then 150 mg collagen was added and kept on magnetic stirrer for 48 h for collagen gel formation. Then, the pH was adjusted to 7.4 using 10M NaOH. The prepared alginate and collagen gels were stored at 4º C until use.

DF-3D bioink preparation

Alginate was dissolved overnight to make 2% Alginate in ddH_2O. To crosslink the Alginate solution, 100 µl of 1-Ethyl-3-(3-dimethylaminopropyl) carbodiimide (EDC) were added (at concentration of 250 mg/ml in ddH_2O) and slowly stirred for 15 minutes at room temperature. Then, 100 µl of 150 mg/ml N-hydroxysuccinimide (NHS) were added, and the solution was stirred for additional 15 minutes. Separately, 3% collagen peptide solution was prepared and mixed with 45% fetal bovine serum (FBS). Alginate and collagen solutions were mixed and stirred for 2 minutes to make a final concentration of 1% Alginate/1.5% Collagen 45% FBS and 5% DMSO.

Alginate/collagen Crosslinking

The EDC was used as a primary crosslinker, which cross links the carboxyl-acid group of Alginate with the primary amines of Collagen. Since EDC-mediated crosslinking is only effective at acidic pH, NHS was used to improve the crosslinking efficiency at pH 7.4. The EDC/NHS-mediated reaction is based on Carbodiimide Crosslinker Chemistry. Then, the pre-hydrogel was treated with $CaCl_2$ to achieve chelation with the carboxyl groups of Alpha-L-guluronic acid (G-block) of alginate. The expected crosslinking reaction is shown in the schematic.

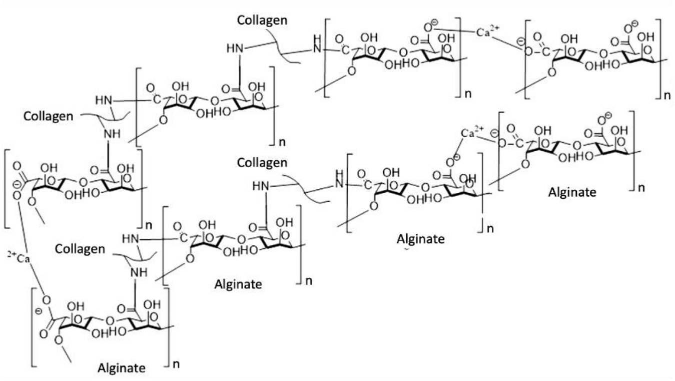

Fourier transform-infrared spectroscopy (FT-IR) analyses of 3D-designed DF-3D bioink

Fourier transform-infrared spectroscopy (FT-IR, model: Nicolet 6700; Thermo Fisher Scientific, USA) was used for the study of bioink composition by determining functional groups of alginate and collagen. For this, freeze-dried bioink samples were used to record the FT-IR data in ATR mode from 4000 to 400 cm^{-1}. The obtained data was compared with pure alginate and collagen.

Rheological properties of DF-3D bioink structures

A discovery hybrid rheometer (model: HR30, TA Instruments, USA), with a cone and plate geometry (Cone angle: 1 degree and truncation gap: 26 µm,) was used to measure the storage and loss modulus at room temperature. After cleaning the plate with DI water, 300 µL of DF-3D bioink was added on the plate for testing. First, amplitude sweep (Oscillation strain sweep: 10^{-2} to 10^{2}) at 1 Hz frequency was carried out to determine the storage modulus and loss modulus values. These data were used to find the viscoelastic properties of bioink and assess recovery of bioink after the transition from a solid-like phase to a more liquid-like phase during extrusion. Then, the shear rate vs. shear stress and shear stress vs. viscosity were measured to determine the properties of the DF-3D bioink under the effect of shear stress.

Fabrication of three-dimensional (3D) structures by DF-3D bioprinting

A custom-made extrusion bioprinter with screw-driven extrusion system, developed in our lab was used for the DF-3D bioprinting. A custom build plate with the reservoir for dry ice was also fabricated to maintain the cryogenic condition. The temperature of the freezing plate was

monitored to be at least below –70 ºC prior to and during printing. To avoid clogging, needle temperature was maintained ~22 ºC using our custom designed print head. A printing speed of 10 mm/s mm was used with a 1/2" blunt stainless-steel nozzle (inner diameter of 250 µm) to create 3D structures based on computer aided design (CAD). Immediately after printing, samples were stored at -80° C for overnight or longer, prior to further processing. For the experiments, cell laden DF-3D bioink samples were then thawed and crosslinked by immersion in culture media prewarmed to 37 °C, and supplemented with 0.05M $CaCl_2$ for 10 minutes, followed by washing with fresh culture media. Thereafter the samples were grown in the culture media at 37° C and 5% CO_2 humidity. Media was changed after 2 hours and again after 24 hours. For cell maintenance, the medium was replaced every three days. For the control samples, scaffolds were prepared at room temperature and directly placed in crosslinking media, and henceforth treated the same as above.

Fabrication of three-dimensional (3D) structures by casting

For casting of 3D structures, DF-3D bioink was injected into water-soluble polyvinyl alcohol (PVA) molds under cryogenic conditions, maintained by dry ice (-78.5 °C). PVA molds were created using FDM printer (S5, Ultimaker) with a 0.4 mm (inner diameter) heated nozzle. PVA molds with DF-3D bioink were stored at -80° C for 24 hours and crosslinked by thawing in culture medium containing 0.05 M $CaCl_2$ at room temperature. Control 3D scaffolds were made by conventional methods, which involves casting without cryoprotectant in a PVA mold at room temperature (22.5º C) (n=6), followed by direct crosslinking in culture medium containing 0.05 M $CaCl_2$ at room temperature for 10 minutes. In creating control samples, no cooling/freezing was involved.

Recovering cell-loaded 3D structures after storage at -80 °C

After validating the designed DF-3D bioink in creating well-defined 3D structures in high temperature cryogenic conditions, we examined the effect of cryopreservation in our DF-3D bioink on multipotent stem cells. Prestained MSCs with Cell Tracker Red CMTPX dye were mixed into our DF-3D bioink, casted into PVA molds, and then flash frozen and stored at -80° C for one week. For cell tracking and distribution, the frozen scaffolds were then immersed in culture media at 37 °C containing crosslinker (0.05M $CaCl_2$) for 10 min, followed by washing with fresh culture

media. Then the samples were incubated in the culture media at 37 °C, 5% CO_2, and 95% humidity. For complete removal of the dissolved PVA mold, media was changed after 2 hours and again after 24 hours. For cell maintenance, the medium was replaced every three days. For the control samples, scaffolds were prepared at room temperature and directly placed in the culture media after crosslinking, and henceforth treated the same as above. After 8 days in culture, viable cells were visualized under a fluorescence microscope using Cell Tracker Red CMTPX dye, which is also retained in daughter cells after replication.

Mechanical properties

To measure the compressive strength of the cryo-fabricated structures, we designed cylindrical scaffolds of 9 mm diameter and 6 mm height. We followed the inequality criterion for maximum nominal compressive strain to determine the adequate dimension for the compression testing of hydrogel scaffolds, as per ISO 604:2002 standard: $\varepsilon_c \leq 0 \cdot 4\,\frac{diameter^2}{length^2}$

The theoretical strain values lie between 0 to 1 (or in 0 to 100%).

Scaffolds were fabricated by casting in a PVA mold, followed by storage at -80 ℃. The next day, scaffolds were thawed in 0.05M $CaCl_2$ culture medium and washed twice with PBS. PBS was changed after 30 min, 2 hours and 24 hours to completely remove the PVA mold (n=5). A dynamic mechanical analyzer (DMA, model: DMA850, TA Instruments, USA) was used to measure the compression properties at 37 ℃ in submersion condition in PBS. Testing was carried out in a rate control-strain ramp mode, at a strain rate of 1% per minute and 0 kN preload.

Scaffold dissolution assay in aqueous media

To determine the stability and integrity of the DF-3D bioink, a dissolution study was conducted. Cylindrical DF-3D bioink scaffolds (9 mm diameter × 6 mm thick) were casted in PVA molds, crosslinked with 0.05M $CaCl_2$, and thoroughly washed with PBS to remove the mold. At least three samples were used per timepoint. Initial weight was measured per each sample and scaffolds were kept in a CO_2 incubator at 37° C for the indicated times. After 7, 14, 21, and 28 days of incubation, the final weight of at least 3 samples was measured per each timepoint. Percent weight loss was calculated as: $[(W_0 – W_x)/ W_0] \times 100$

W_0 - Initial sample weight, W_x – Final sample weight at time X.

Swelling assay

The swelling behavior of DF-3D bioink scaffolds was studied in PBS at 37°C. Crosslinked samples (with 0.05M $CaCl_2$) were freeze-dried, and weight was measured (Wd). Then, samples were immersed in PBS. Samples were removed and weight was measured (Wt) every 5 minutes for 30 minutes, then every 30 minutes. Degree of swelling (swelling ratio), swelling rate, equilibrium swelling ratio, and percentage equilibrium liquid content were calculated using the following equations [32, 51].

$$\text{Swelling ratio } = \frac{W_t - W_d}{W_d} = \text{ degree of swelling} \quad \text{...........}(1)$$

$$\text{Swelling rate } = weight\ change\ per\ unit\ time = \frac{W_{t+\Delta t} - W_t}{\Delta t} \quad \text{...........}(2)$$

$$\text{Equilibrium swelling ratio } = \frac{W_{equ} - W_d}{W_d} \quad \text{...........}(3)$$

$$\text{Percentage equilibrium water content } = \frac{W_{equ} - W_d}{W_{equ}} \times 100 \quad \text{...........}(4)$$

W_t, is the weight of swollen samples at time t, W_d is the weight of dried samples, $W_{t+\Delta t}$, is the weight of the swollen samples at time interval Δt, and W_{equ} is the weight of swollen samples at equilibrium state.

Scaffold shape fidelity

To study the shape fidelity, 3D structures were created by extrusion as well as by casting of DF-3D bioink without cells at high cryogenic temperatures (-80° C), maintained by dry ice. After fabrication, frozen 3D structures were stored at -80 °C for 24 hours. Frozen structures were thawed with 0.05 M $CaCl_2$ at room temperature and were examined for dimensional deviation from the computer-aided design (CAD) to assess the efficacy in creating well-defined structures with high fidelity and reproducibility.

Assessment of Interlayer mixing during DF-3D bioprinting

For interlayer mixing study and calibration, multilayered structures were created using DF-3D bioink (without cells) stained in different colors (**Supplement Figure 2**). After 24 hours of storage at -80 °C, frozen structures were thawed in 0.05 M $CaCl_2$ at room temperature, washed and examined for interlayer mixing.

Microscopic interlayer mixing of cell laden DF-3D bioink

To assess microscopic interlayer mixing (**Figure 3**), cells (Tu167) were pre-stained with either red fluorescence (Cell Tracker Red CMTPX) dye, or green fluorescence dye (Green CMFDA Invitrogen) according to the manufacturer protocol (Thermo Fisher Scientific), prior to incorporation into the DF-3D bioink. Cells were hand printed using syringes containing red, blue or unstained DF-3D bioink in –80º C, thawed and examined for interlayer mixing under EVOS FL florescence microscope. To further investigate the mixing of cells at the layer interface in the printed 3D structures, MSCs were pre-stained in red (Red CMTPX, Invitrogen) and green (Green CMFDA, Invitrogen). The pre-stained cells were mixed in the DF-3D bioink and printed manually to create multilayered structures at Cryogenic temperature. After 24 hours of storage at -80 ℃, these frozen structures were thawed in culture media containing 0.05 M $CaCl_2$. These structures were then observed under EVOS FL fluorescence microscope to determine interlayer mixing.

Cell culture of multipotent stem cells (MSCs)

MSC lines were maintained in complete medium: DMEM (Invitrogen) 10% FBS (Takara) and penicillin/streptomycin (50 U/50 μg/ml; Invitrogen), supplemented with 0.1 mM non-essential amino acids (NEAA; Invitrogen), 1 mM sodium pyruvate (Invitrogen), 2 mM GlutaMAX (Invitrogen), and 0.1 mM beta-mercaptoethanol (Life Technologies/Gibco), as we previously described [29, 30]. Cell culture medium was changed every 3 days and cells were routinely passaged at 70% confluency every 4-7 days, using Accutase (EMD Millipore). The institutional review board (IRB) protocol was approved with informed consent by the University of Maryland, Baltimore institutional review board (IRB protocol #HP-00062781-1).

Osteogenic differentiation medium

To induce differentiation toward bone, osteogenic media was prepared as previously described using phenol red free DMEM (Life Technologies), supplemented with 150 µM L-ascorbic acid 2-phosphate (Sigma), 10 mM b-glycerophosphate (Sigma), 10 nM dexamethasone (Sigma), 1% penicillin and streptomycin (Life Technologies) and 10% Fetal Bovine Serum (FBS).

Osteogenic differentiation of MSCs grown inside the 3D scaffolds

For osteogenic differentiation, alginate-collagen DF-3D bioink was loaded with MSC cells and casted in or infused into PVA molds at cell density of 2.9×10^6 cells/ml and stored at -80 ℃ for at least 24 hours, followed by thawing in a culture media containing 0.05 M $CaCl_2$, pre warmed to 37 ℃, for 10 minutes. Next, cell-laden scaffolds were washed in fresh culture media and incubated for 30 minutes. The media was replaced after 2 hours and again after 24 hours and kept at 37 ℃ in a 5% CO_2 incubator at 95% humidity. On the third day, the media was changed with osteogenic differentiation medium, while controls were replenished with fresh culture media. Media was changed twice a week throughout the study.

Live cell quantification

For Live cell quantification, cells were stained with CellTracker™ Red CMTPX Dye (Invitrogen™; Fisher Scientific). Which can diffuse through the membranes and turn into red fluorescence in live cells. Then, the red and unstained cells were counted manually under a fluorescence microscope at Z plane intervals of 8-μm. Unstained cells were considered dead. Percentage of live cells was then determined by calculating the ratio of red cells to the total number of cells (the sum of CMTPX positive and negative dead cells). Please also note our live/dead data on Figure 6C, D. Both live (green) and dead cells (red) were counted, then, the percent of live cells was calculated compared to the average total number of cells per sample (in 4 biological replicates in independent experiments). In figure 9, since hardly no red dead cells were found, to count the total number of cells, cells were co-stained with Hoechst staining (blue).

Live/Dead cell viability assay

Cell viability was measured by using Live/Dead kit (Invitrogen), according to the manufacturer protocol (n=3 / group). Imaging was done under inverted EVOS FL fluorescence microscope. Cell viability was quantified by counting live (green) and dead (red) cells using ImageJ software [28, 52].

Effect of $CaCl_2$ concentration and crosslinking time on cell viability

Crosslinking conditions were optimized to maintain maximal cell viability and crosslinking efficiency (**Supplement Figure 2**). MSCs (2.5×10^4 cells/ml) were incubated at a range of $CaCl_2$ concentrations (0.05M, 0.1M, 0.2M, 0.5M, 0.8M, and 1M) for various time points (10, 30 minutes,

1 and 5 hours), followed by viability assay (LIVE/DEAD™ cell imaging kit, Invitrogen), according to the manufacturer protocol.

Cryo-SEM (Cryo-Scanning Electron Microscope) of cell-laden DF-3D bioink

To study DF-3D bioink, a cryo-scanning electron microscope, was used for microstructural analyses. Scaffolds were fixed in 10% formaldehyde and sliced in to ~1 mm thin section and adhered to an aluminum stub using cryoembedding solution. Thin sections were frozen in slush nitrogen to avoid Leidenfrost effect. The surface of frozen samples was fractured and sublimated to expose the inner microstructure. Thereafter, samples were gold-coated (to avoid image distortion due to charge accumulation on sample surface during imaging) and were observed under a cryo-SEM (Quanta 200, FEI, USA), equipped with cryo-transfer unit (model: ALTO2100, Gatan, USA), at -130 ℃ and operated at an accelerating voltage of 5 kV and a working distance of 6 mm.

Preparation of Cryosections

DF-3D bioink scaffolds with or without cells were fixed in 10% formaldehyde for 16 hours at 4 ℃, cryo-embedded and 14 µm thick cryosections were produced using cryo-microtome by slicing parallel to the vertical axis of cylindrical scaffolds. The sections were transferred on a glass slide and were stored at -80 ℃ until use,

Hematoxylin and Eosin (H&E)

For H&E staining, frozen sections were baked at 40 ℃ for 20 min, followed by cooling to room temperature for 5 minutes. Samples were then stained in hematoxylin (Sigma Aldrich, USA) for 3 minutes then washed with 5 changes of tap water until no blue color was present and then washed once with DI water. Slides were counterstained with Eosin (Sigma Aldrich, USA) for 2 minutes and then rinsed by dipping in tap water and mounted by coverslip using prior to imaging.

Von Kossa staining

For Von Kossa staining, frozen sections were baked at 40º C for 20 minutes and cooled to room temperature. Next, slides were fixed in 10% formaldehyde for 30 minutes, washed with 1×PBS and then with DI water. Sections were then kept in 5% silver nitrate solution (in DI water) at room temperature and placed under ultraviolet light for 3 hours. Next, the slides were washed in DI

water. Unreacted silver was removed by incubation in 5% sodium thiosulfate at room temperature. Slides were rinsed three times in DI water and counterstained with nuclear fast red for 5 minutes. Slides were rinsed in running tap water, followed by wash DI water. Then, slides were briefly dehydrated in absolute ethanol and covered by coverslip using permanent mounting medium. Phase contrast microscope was used for imaging.

Alizarin Red Staining

For Alizarin Red staining, 2% Alizarin Red S reagent was prepared by dissolving 1g Alizarin Red S powder (Sigma) in 50 ml DI water, pH was adjusted to 4.2, and the solution was filtered through 0.22um SteriFlip (Millipore) filter. Next, slices were rinsed gently in DI water, and stained in the Alizarin Red S solution for 20 minutes at room temperature. After decanting the staining solution, the slices were washed 5 times in DI water until no more staining was drained our of the scaffolds, and taken for microscope imaging.

Immunofluorescence staining

Slides were hydrated, blocked in 1% BSA, 10% fetal bovine serum, and 0.2% Tween 20, and incubated at 4°C overnight with the primary antibodies: mouse anti-RUNX2 (1:100, Santa Cruz), rabbit anti-COL1A1 (1:100; Millipore), rabbit anti-Osterix (1:200, Millipore), goat anti-RANKL (1:200, Santa Cruz), mouse anti-RANKL (1:200, Santa Cruz) and goat anti-ALP (1:200, R&D Systems) in blocking solution. Nuclei were stained with DAPI (Roche Life Sciences). Undifferentiated cells were grown in similar conditions and cells stained without primary antibody, were used as controls. Samples were visualized with fluorescent Alexa568, Alexa488 or Alexa647 secondary antibodies (Invitrogen), under a Nikon CSU-W1 Spinning disk field scanning-confocal microscope system, or a Leica DMi8 florescence microscope.

Micro-CT (Micro Computed Tomography) of cell-laden scaffolds

To assess osteogenic activity for bone-like mineral deposition in the 3D scaffolds. MSC-laden scaffolds were fixed in 10% formaldehyde and analyzed using a Skyscan 1172 micro-CT. During the analyses, a polystyrene holder was used, and scaffolds were submersed in PBS. Data were collected at room temperature at 55 kV and 188 μA using 0.5 mm aluminum filter and 1° step size.

Statistical analyses

All data are shown as the mean±S.E.M of multiple independent experiments, with independent biological replicates. Student's t-test or one-way ANOVAs (when appropriate) were used for statistical analyses. Significant interactions were followed by Tukey or Fisher LSD post-hoc comparisons when appropriate. Statistical analyses and figure generation were performed with GraphPad Prism 8.1.0 software.
